# Telomere Length as a Marker of Biological Age: State-of-the-Art, Open Issues, and Future Perspectives

**DOI:** 10.3389/fgene.2020.630186

**Published:** 2021-01-21

**Authors:** Alexander Vaiserman, Dmytro Krasnienkov

**Affiliations:** Laboratory of Epigenetics, D.F. Chebotarev Institute of Gerontology, Kyiv, Ukraine

**Keywords:** leukocyte telomere length, age-related telomere shortening, telomerase, biomarker of biological age, aging-related disease, mortality

## Abstract

Telomere shortening is a well-known hallmark of both cellular senescence and organismal aging. An accelerated rate of telomere attrition is also a common feature of age-related diseases. Therefore, telomere length (TL) has been recognized for a long time as one of the best biomarkers of aging. Recent research findings, however, indicate that TL *per se* can only allow a rough estimate of aging rate and can hardly be regarded as a clinically important risk marker for age-related pathologies and mortality. Evidence is obtained that other indicators such as certain immune parameters, indices of epigenetic age, etc., could be stronger predictors of the health status and the risk of chronic disease. However, despite these issues and limitations, TL remains to be very informative marker in accessing the biological age when used along with other markers such as indices of homeostatic dysregulation, frailty index, epigenetic clock, etc. This review article is aimed at describing the current state of the art in the field and at discussing recent research findings and divergent viewpoints regarding the usefulness of leukocyte TL for estimating the human biological age.

## Introduction

The last century has been characterized by an unprecedented rise in human life expectancy across the globe. This demographic trend, however, was generally not accompanied by the same extension in healthspan and productivity (Lee et al., 2020). This is because aging *per se* is the major risk factor for most pathological conditions that limit healthspan and promote chronic disorders affecting the elderly, including immuno-senescence, cardio-metabolic disorders, osteoporosis, sarcopenia, arthritis, cataracts, neurodegenerative diseases and most cancers (Franceschi et al., 2018). Therefore, development of effective tools for slowing down the aging rate is currently a priority agenda for research organizations and for health policy makers across countries (Yabluchanskiy et al., 2018). In this context, an important task is the development of effective tools to evaluate the rate of aging both in individuals and in groups of patients participating in clinical trials specifically targeting aging and related multimorbidity (Ferrucci et al., 2020; Moskalev, 2020). Historically, functional tests such as grip strength, walking speed, chair rising, and standing balance times were most commonly used in such studies (Ferrucci et al., 2016; Justice et al., 2016). These tests, however, are too sensitive to transient changes in health status caused by non-chronic diseases such as colds and indigestion, so they can hardly provide a reliable estimate of the aging rate *per se*. Therefore, considerable efforts were made over the past few decades to develop measures of biological aging which are reliable, precise, and robust. Among them, there are parameters characterizing aging-related processes such as genomic instability, cellular senescence, DNA methylation, proteostasis, and mitochondrial function (Jylhävä et al., 2017; Ferrucci et al., 2020). None of the above measures, however, represent an exhaustive measure of biological age.

According to the American Federation for Aging Research (2016), a true biomarker of aging should meet the following criteria:

1. predict remaining life expectancy better than chronological age,
2. monitor mechanisms underlying the aging process but not a specific disease,
3. be subject to repeated tests without harming the individual,
4. be testable in both laboratory animals and humans.

Telomere length (TL) possesses many properties that make it suitable to be used as a biomarker of aging. Telomeres of vertebrates represent repetitive (TTAGGG)n sequences located at the ends of linear chromosomes. These chromosomal structures are known to be crucially involved in the process of cellular senescence (Victorelli and Passos, 2017) and their age-associated shortening is commonly regarded as an important contributor to organismal aging (McHugh and Gil, 2018). Therefore, TL is currently recognized by many authors as powerful biomarker of aging and aging-associated pathological conditions (Fasching, 2018). The advantages of TL in order to be a biomarker of aging include a correlation with chronological age throughout the entire life course, predictive power for the disease condition and mortality and also large responsiveness to either adverse or beneficial exposures (Hastings et al., 2017). Moreover, it is recognized as an attractive biomarker candidate to track changes in the human aging rate over the course of intervention trials designed to delay the onset or retard the progression of aging-associated pathological conditions. However, while TL satisfactorily meets the criteria 3 and 4 of the American Federation for Aging Research due to an opportunity to a minimally invasive, repeated testing without harm to human subjects and a testability in both laboratory animals and humans, its compliance with the criteria 1 and 2 is rather questionable (Sanders and Newman, 2013).

Presently, peripheral blood leukocyte TL (LTL) is often identified in cross-sectional and longitudinal analyses when certain patient cohorts are compared to age- and sex-matched individuals. However, even though convincing evidence has been obtained from *in vitro* and *in vivo* models that TL reflects levels of both the cellular senescence and chronic disease-related oxidative stress, epidemiological and clinical findings are far less conclusive (Sanders and Newman, 2013). Indeed, a large inconsistency was observed among studies. Such a discrepancy could be, at least partly, attributable to differences in the methods of TL measurement and statistical modeling used, variations among investigated populations, etc. This inconsistency was, however, so great that some authors have even questioned whether the link between TL and aging-associated processes really exists and, consequently, it has led to hot debates about whether TL is really a reliable and valid tool to evaluate the rate of aging in human populations (Sanders and Newman, 2013). Moreover, the phenomenon of the age-related telomere shortening is extremely complex and biological mechanisms underlying this process are not yet definitively established. In particular, it remains still unclear whether telomeric aging reflects a mitotic clock-like process or it is rather a biomarker of stress or a biological mechanism that transfers stress-associated signals to the cell (Koliada et al., 2015; Notterman and Schneper, 2020). If the latter, telomere shortening can be rather a proxy for life-course stress exposures than a genuine cause of aging (von Zglinicki and Martin-Ruiz, 2005). Nevertheless, despite this uncertainty, TL currently remains one of the most widely used biomarkers of aging in epidemiological and clinical studies. In recent years, TL is also being increasingly used as a potential biomarker in personalized medicine (Gorenjak et al., 2018).

The present review is aimed at discussing diverging views and research findings regarding the usefulness of TL for estimating the human biological age.

## Basic Principles of Telomere Biology

Telomeres play vital roles in multiple cellular processes because they protect chromosomes from end-to-end fusions and chromosomal instability (Aksenova and Mirkin, 2019). The repetitive TTAGGG sequences that constitute telomeric DNA are bound by the protective protein complex, Shelterin. This complex, together with proteins involved in chromatin remodeling, shapes the telomere structure thereby protecting chromosome ends (Tomita, 2018). Two key telomere features are the formation of DNA loops at chromosome ends (T-loops) and the transcription of telomeres producing G-rich RNA (TERRA). In the t-loop structure, the 3′ end of the G-rich strand protrudes as a single stranded overhang, known as the G-overhang (Turner et al., 2019). This G-strand overhang loops back to form a t-loop and invades the 5′ double stranded telomeric duplex, thereby forming so-called D-loop. This structure ensures that loose DNA ends are housed internally within the nucleoprotein structure (Turner et al., 2019). The formation of such looped structures is an important mechanism that protects telomeres from premature degradation. Despite their heterochromatic state, telomeres are able to be actively transcribed resulting in a production of long non-coding RNAs called TERRA (telomeric repeat-containing RNA). TERRA molecules play crucial role in telomere biology, including regulation of telomerase activity and formation of heterochromatin at chromosome ends (Bettin et al., 2019; Lalonde and Chartrand, 2020).

At each somatic cell division cycle, telomeres shorten by 50–200 bp through incomplete synthesis of the lagging strand during the DNA replication (Srinivas et al., 2020). This is due to inability of DNA polymerase to completely replicate the 3′ end of the DNA strand (a phenomenon commonly referred to as “the end-replication problem”) (Watson, 1972; Olovnikov, 1973). Moreover, since the G-rich telomere repeat sequence is known to be highly susceptible to oxidative damage (Oikawa and Kawanishi, 1999), telomeres may be directly damaged by oxidative stress, thereby driving the cell into senescence (Barnes et al., 2019). Considering this, it has been recently suggested that telomere-induced senescence of post-mitotic cells might be a key driver of aging (von Zglinicki et al., 2020).

In culture, somatic cells have limited replication potential reaching a time point at which cell division ceases. This time point is characterized by shortening (“attrition”) of particular telomeres to a critical size incompatible with their functioning, thereby resulting in cell cycle arrest and cellular senescence. Therefore, TL is suggested to limit the cell division number and acts as a “mitotic clock” in the cell (Olovnikov, 1996), and telomere shortening may cause decreasing of proliferative potential and be a marker for cellular senescence (Liu et al., 2019a). In a multicellular organism, TLs are highly heterogeneous across different tissues and cell types depending, at least partly, on the tissue-specific proliferation rate, but they generally tend to decrease with age in all proliferating tissues (Demanelis et al., 2020).

**Figure 1:**
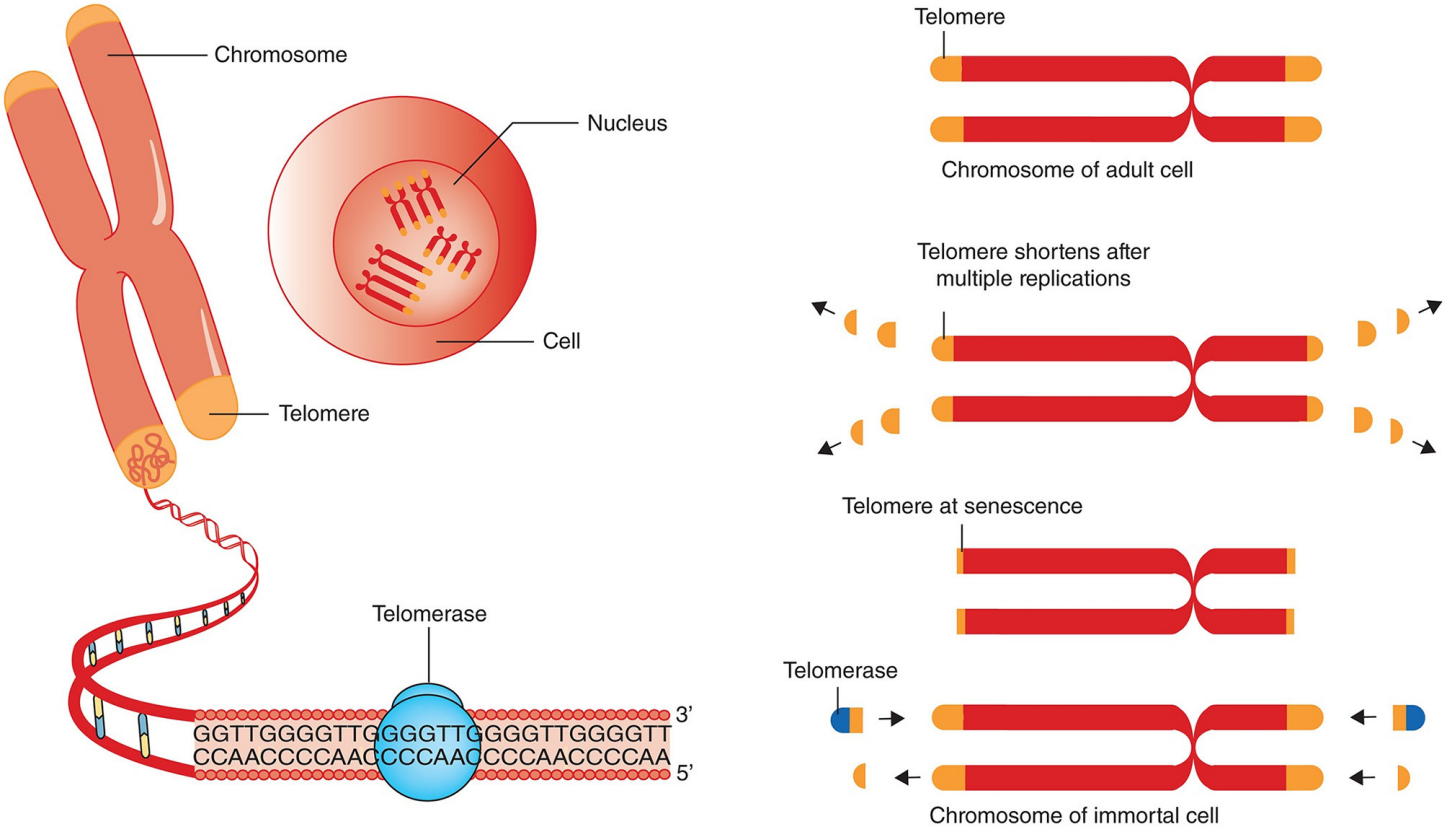
Telomere attrition, telomere length and telomerase. Chromosomes have repeated base segments called telomeres that shorten with each replication cycle (cell division). The enzyme telomerase has the capability to extend the telomere ends, thus prolonging cell life and potentially inducing immortality (which is a cancer cell hallmark). The figure and its legend are reproduced from the article by Aunan et al. (2016) with permission from John Wiley and Sons (License Number 4938770663443). Copyright © 2016 BJS Society Ltd Published by John Wiley & Sons Ltd.

The size of critically short (“uncapped”) telomeres may be stabilized by telomerase, a reverse transcriptase enzyme that can elongate chromosome ends *de novo*. Two main components of human telomerase are telomerase reverse transcriptase (TERT) and telomerase RNA component (TERC) serving as a template for the telomere elongation (Rubtsova and Dontsova, 2020). In humans, this enzyme is known to be expressed during early *in-utero* development, is inactivated in most adult cells except for the germ line and embryonic stem cells and immune cells, and reactivated in the majority of cancers (Shay and Wright, 2019). Telomerase was shown to be insufficient to maintain normal TL even in proliferating stem cells that may express it; therefore, gradual shortening of telomeres also occurs in these cells (Lai et al., 2018; Celtikci et al., 2020) (Figure 1). Since most human somatic cells have low or no telomerase activity, it results in age-related telomere erosion and associated pathological processes. Therefore, activation of telomerase is regarded by several authors as promising therapeutic modality in the treatment of degenerative aging disorders (Bernardes de Jesus and Blasco, 2011; Prieto-Oliveira, 2020). However, even although telomerase indeed has potential in anti-aging medicine, the fact that it is over-expressed in about 90% of human cancers raises doubts about the applicability of telomerase activators in clinical practice (Smith-Sonneborn, 2020).

## Genetic and Environmental Determinants of TL

TL and the rate of age-related telomere shortening depend on both genetic and environmental factors. In twin studies, high heritability of TL was demonstrated and many specific loci related to TL were identified. For example, in the study by Hjelmborg et al. (2015), the heritability of LTL at baseline was estimated at 64%; the heritability of age-dependent LTL attrition rate was less (28%) but also significant. Genetic mutations related to short TL have been found to lead to diseases such as pulmonary fibrosis, dyskeratosis congenita, and also some other clinical conditions commonly grouped under the term “telomere syndrome” (El-Chemaly et al., 2018). A relative contribution of genetically determined TL to the risk of various neoplastic and non-neoplastic disorders was repeatedly evaluated by a Mendelian Randomization approach (a research strategy aimed at evaluating causality in observational epidemiological studies). Mendelian Randomization studies have indicated that long genetically predicted TLs (gTLs) are commonly associated with greater risks for several cancers, whereas short gTLs are associated with increased risks of some age-related degenerative diseases, including cardiovascular disorders (CVDs) and Alzheimer's disease (Telomeres Mendelian Randomization Collaboration et al., 2017; Protsenko et al., 2020). Recenely, a potential causal link between short gTLs and accelerated facial aging was demonstrated using Mendelian randomization analysis in participants of the UK Biobank data (Zhan and Hägg, 2020).

**Figure 2:**
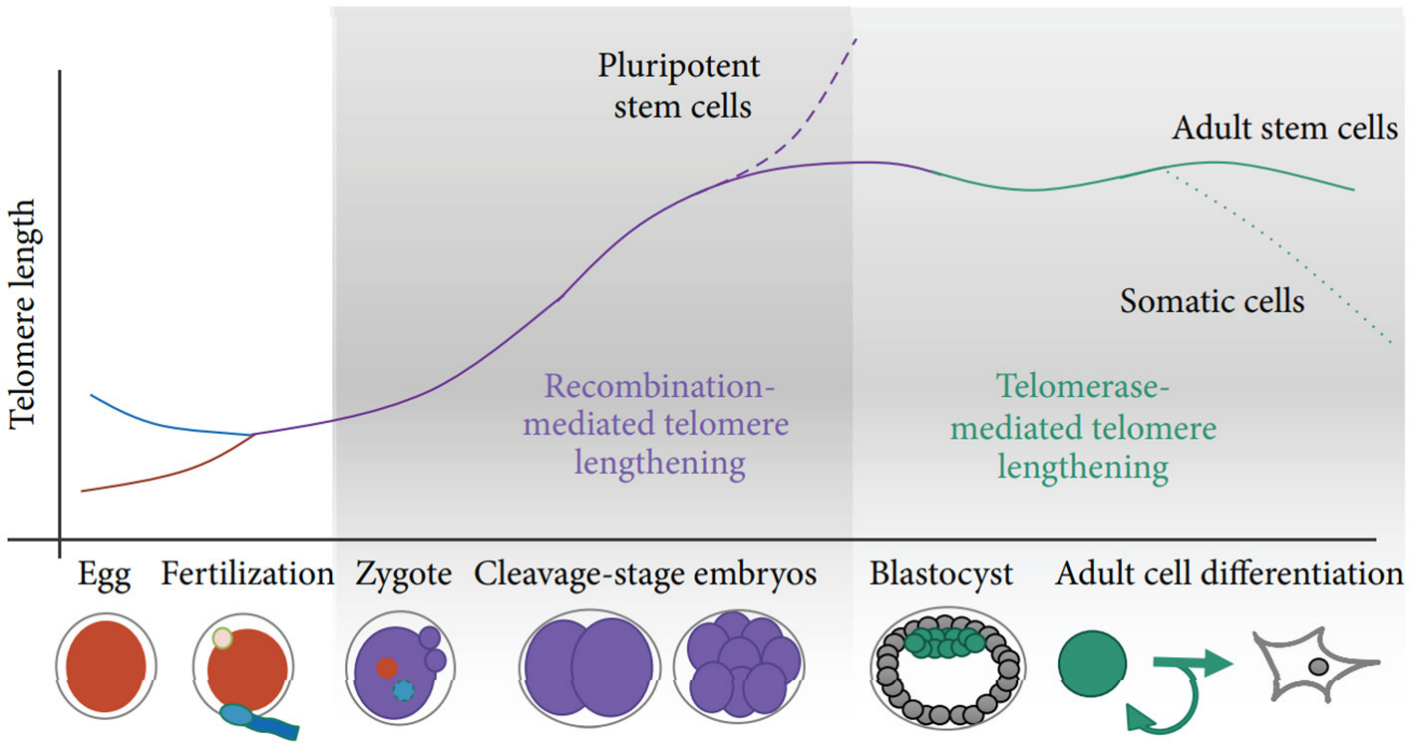
Schematic of telomere length reprogramming in mammalian embryonic development. The greatest telomere lengthening takes place during the earliest stages of preimplantation development, the cleavage-stage embryo, which may coincide with zygote genome activation. Recombination-mediated telomere lengthening (ALT) is also purportedly responsible for reprogramming in pluripotent stem cells, including ESCs, ntESCs, and iPSCs. Later, in development and adult life, telomerase becomes the dominant telomere maintenance mechanism for the inner cell mass and in tissue-specific telomere replenishment in stem cell niches. The figure and its legend are reproduced from the article by Kalmbach et al. (2014) distributed under the terms of the Creative Commons Attribution License. Copyright: © 2014 Keri Kalmbach et al.

Two potential sources of TL heritability include inherited variation in non-telomeric regions (e.g., single nucleotide polymorphisms affecting telomere maintenance) and TL variability in gametes which produce zygotes (the “direct” inheritance). Strong evidence has been provided that TLs in parental germ cells may impact TL in offspring cells thereby contributing to heritability of TL (Delgado et al., 2019). Indeed, TL was shown to increase with age of father in sperm (correspondingly, descendants of older fathers inherit longer telomeres), while higher maternal age at conception appears to be associated with shorter offspring TL (Eisenberg and Kuzawa, 2018; Eisenberg et al., 2019). Remarkably, the impact of parental germ cells on TL remains significant even despite the telomere “reprogramming” throughout embryonic development, initially by the recombination-based mechanisms and then through the activity of telomerase at the blastocyst stage and later (Kalmbach et al., 2014, see also Figure 2 for schematic illustration).

Multiple findings indicate that TL may be also significantly modulated by environmental and life-style factors throughout the life course. Among important factors affecting life-course TL dynamics, there are developmental experiences such as intrauterine unfavorable events (Entringer et al., 2018; Habibi et al., 2020), TL at birth (Gorenjak et al., 2020), as well as early life adversity (family's low socioeconomic status, childhood neglect or abuse, etc.) (Belsky and Shalev, 2016; Ridout et al., 2018; Beijers et al., 2020). This is all the more important that early-life exposures can likely affect health status and longevity not only in directly exposed individuals but also in their offspring (Vaiserman et al., 2017). Recently, evidence was obtained that TL may be substantially involved in such trans-generational effects. According to a hypothesis recently proposed by Epel (2020), TL can be inherited not just through classical genetic mechanisms but also through direct (epigenetic-like) transmission of the parental germ-line TL. More specifically, if parental gametes are vulnerable to stress, then chromosomes, together with their telomere sequences which were shortened in consequence of stress exposure, can be directly transmitted to offspring thereby affecting health outcomes and longevity in subsequent generations. Adult-life exposures to environmental factors such as infection (Ilmonen et al., 2008), psycho-emotional stress (Epel et al., 2004; Boccardi and Boccardi, 2019; Mayer et al., 2019), nutrition (Canudas et al., 2020; Galié et al., 2020), physical activity (Semeraro et al., 2020), smoking (Astuti et al., 2017), alcohol consumption (Dixit et al., 2019), marital status (Chen et al., 2020), therapeutic interventions (Srinivas et al., 2020), etc. were also shown to have long-term impacts on TL.

In conclusion, better understanding of these processes can likely lead to developing novel treatment strategies specifically targeted at reversing damaging effect of stress on telomeres (Shalev and Hastings, 2019; Epel, 2020) and thereby to promote human healthspan.

## Life-Course Dynamics of TL

An inverse correlation between TL and human chronological age is well-documented in the literature. For instance, in a systematic review of such a relationship in adults, a significant negative correlation of about −0.3 between mean chronological age and mean LTL was found across 124 cross-sectional studies (Müezzinler et al., 2013). This relationship, however, is not linear and depends on the stage of the human life cycle. Following fertilization, TL was shown to decrease up to the stage of embryo cleavage and, thereafter, to increase up to the blastocyst stage (Turner et al., 2010). These changes are in line with changes in telomerase activity during this time (Wright et al., 2001). At the stage of blastocyst, both telomerase activity and TLs are again substantially increased. The gestational dynamics of TL has been determined in few studies only, and results of these studies are contradictory. A decrease in TL and telomerase activity throughout 6–11 weeks of human gestation was observed by Cheng et al. (2013); after that, they both remained rather constant until birth. In the study by Sorochynska et al. (2018), however, both TL and telomerase activity were shown to increase between 5 and 10 weeks of gestation. After that, they reached a plateau and remained constant up to gestational week 12.

In newborns, TLs appear to be associated with parental TLs, though controversy exists regarding the relative maternal and paternal contributions (Eisenberg, 2014; Turner et al., 2019). After birth, telomeres are steadily shortened with age. The rate of telomere attrition was shown to vary throughout a human lifetime (Turner et al., 2019). It is much more pronounced during first two years of life (throughout the period of rapid somatic growth) than during later life (Frenck et al., 1998; Zeichner et al., 1999). It has been also found that those individuals who have either shorter or longer TL in their childhood, compared to the average population value, tend to maintain this rank throughout the rest of the life course (Benetos et al., 2013). In other words, those individuals who enter their adult life with either short or long telomeres are likely to have short or long telomeres, respectively, during the remaining lifetime. This indicates that inter-individual variation in TL is established mainly early in life and that early life is an important stage in determining TL and may have a lasting effect on TL throughout the entire life course (Factor-Litvak and Susser, 2015). Thereby, at any age point, the resulting TL is a joint function of the newborn TL setting and of the TL attrition rate over the lifetime (Entringer et al., 2018). Interestingly, findings from some studies indicated that rate of age-dependent telomere shortening during adulthood was more pronounced in those persons who had longer telomeres at baseline (Turner et al., 2019). Several authors suggest that the effect of the dependency of age-related LTL attrition on the baseline LTL can be explained by a well-recognized statistical phenomenon such as a “regression to the mean” (Verhulst et al., 2013). Such an effect occurs when individuals who are measured with an extreme error (no matter negative or positive) at baseline, will on average tend to be measured with a much smaller error at follow-up (Barnett et al., 2005). In analyzing the potential role of this statistical artifact in age-dependent TL dynamics, Verhulst and co-authors revealed modest effect which, however, remained statistically significant even when correcting for the “regression to the mean” effect, indicating that high baseline TL is really associated with higher rate of age-related TL attrition (Verhulst et al., 2013).

**Figure 3:**
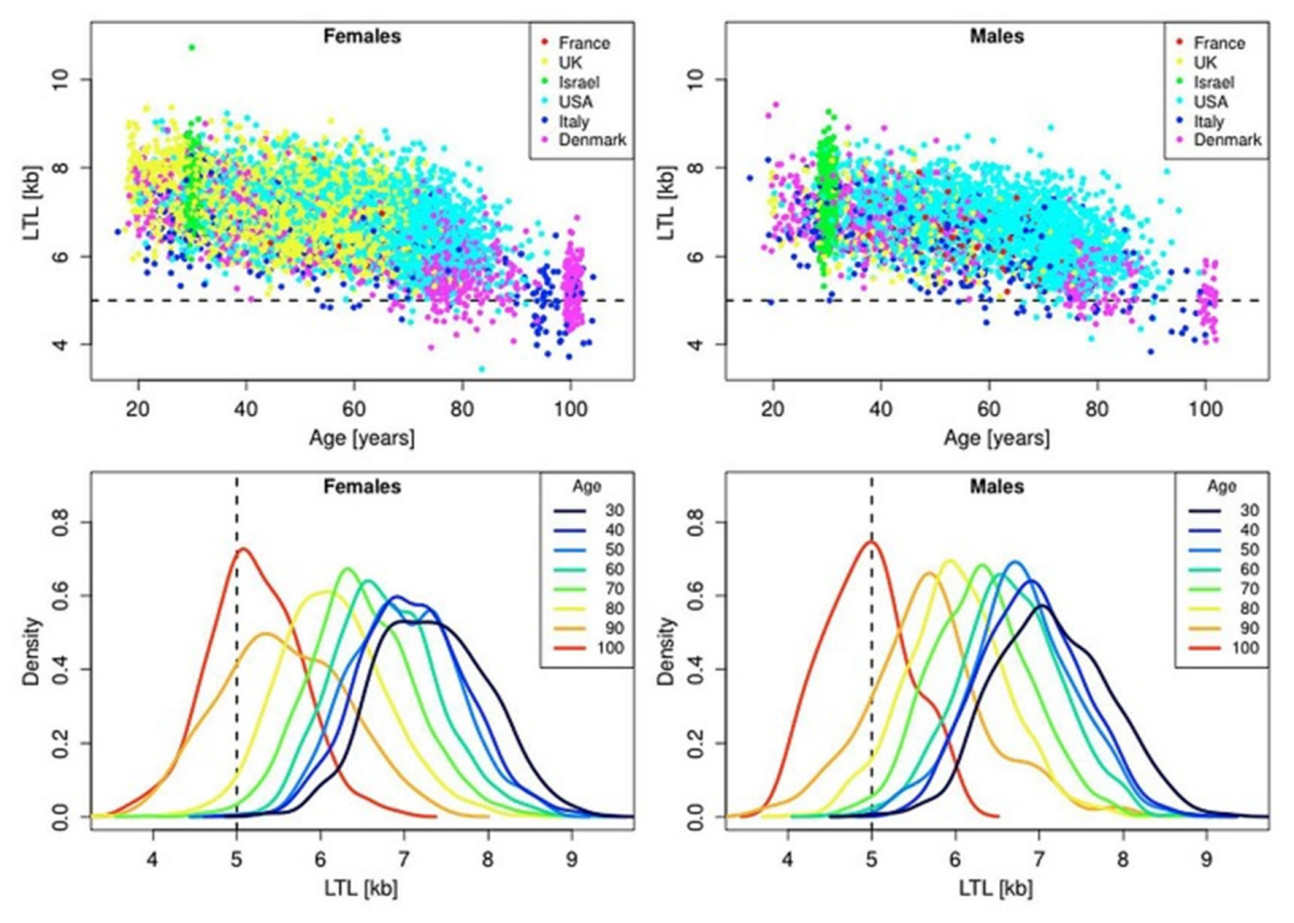
Scatter plots and density plots of LTL as a function of age for males and females residing in different countries. Measurements of LTL were performed in the same laboratory on DNA donated by participants in different studies in different countries. The horizontal dashed lines in the top panels and vertical dashed lines in the bottom panels indicate LTL values of 5 kb. The bottom plots are smoothed histograms obtained by kernel density estimation. The figure and its legend are reproduced from the article by Steenstrup et al. (2017) distributed under the terms of the Creative Commons Attribution License (CC BY 3.0). Copyright © 2017 Steenstrup et al.

In the blood from healthy newborns, median leukocyte TLs (LTLs) ranged from 8.5 to 13.5 kb in the Okuda et al. (2002) study and from 7.0 to 11.6 kb in the Factor-Litvak et al. (2016) study. With age, LTL was found to shorten with an average annual rate of 30–35 bp (Herrmann et al., 2018), reaching about 5–6 kb in people over 60 years old (Calado and Dumitriu, 2013). This appears to be an important point in the context of current debates on whether the human longevity has a maximal natural limit. Steenstrup et al. (2017) suggested that LTL of 5 kb is the “telomeric brink,” which denotes a high risk of impending death. Such a telomeric brink can be likely reached, according to the authors, within the present-day life expectancy and, a fortiori, within the 100-years life expectancy (see Figure 3 for illustration). If so, then current upward trend in life expectancy may confront a biological limit because of crossing the telomeric brink. Since longer TLs are beneficial for a healthy aging (Boccardi and Boccardi, 2019), this feature of telomere biology is apparently vital for the normal functioning of aging organism.

**Figure 4:**
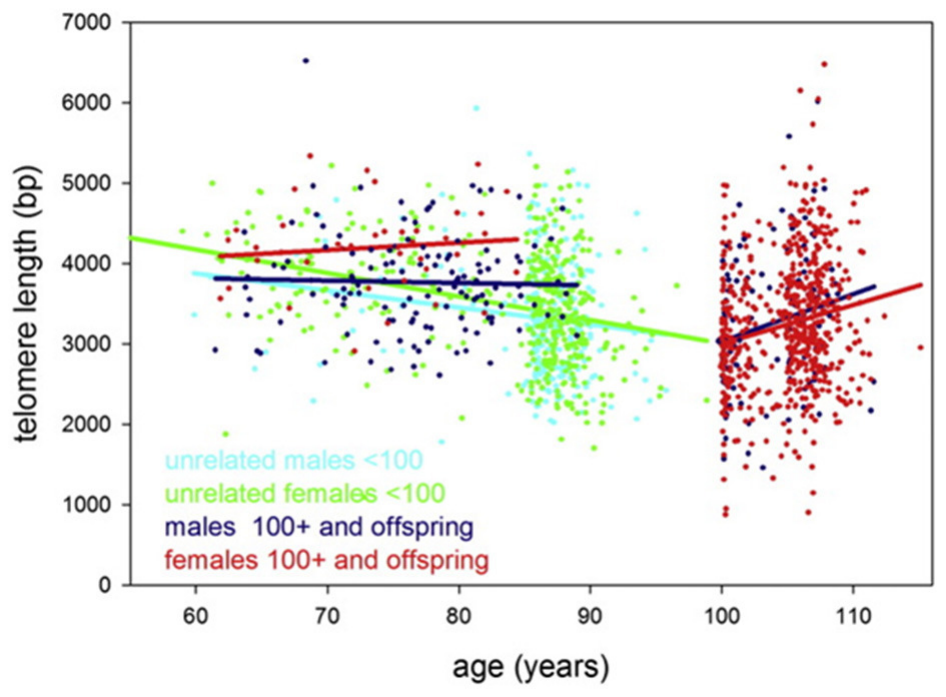
Telomere length in study participants up to 115 years of age. Leukocyte telomere length vs. age is shown for males (blue or cyan) and females (green or red). Centenarians, (semi)supercentenarians, and centenarian offspring are shown in blue (males) or red (females), respectively. Unrelated participants younger than 100 years are indicated in cyan (males) or green (females). Regression lines belonging to these groups are indicated by the same color. The figure and its legend are reproduced from the article by Arai et al. (2015) distributed under the terms of the Creative Commons CC-BY license. Copyright © 2015, Elsevier.

It should be taken into account, however, that findings from some studies contradict the prediction of Steenstrup and co-authors. For example, in the study by Arai et al. (2015), the average TL values dropped to about 3.5 kb in centenarians and then remained approximately unchanged or, paradoxically, even enlarged in supercentenarians compared to younger age groups. More specifically, TLs of unrelated persons were found to shorten by ~21 ± 8 (males) and 29 ± 4 (females) bp/year, while after 100 years of age, TLs were found to increase by 59 ± 25 (males) and 48 ± 11 (females) bp/year (Figure 4). Evidence was obtained that centenarians can maintain TL and telomerase activity better than non-centenarians, and that healthy centenarians have significantly longer telomeres than unhealthy centenarians (Terry et al., 2008; Tedone et al., 2014, 2019). This could be likely explained by preserved telomerase activity in several cell lines. For example, stimulated T cells from centenarians expressed higher telomerase activity levels and they proliferated better than T cells from younger unrelated 67- to 83-year-old individuals (Tedone et al., 2019). The paradoxical ≪elongation≫ of TL in centenarians may likely be explained by a survivor effect (Mather et al., 2011). Indeed, if individuals with shorter TLs are more susceptible to age-associated pathologies and, consequently, are at higher risk of mortality, then these individuals will tend to die earlier. Therefore, in cross-sectional studies of oldest-old participants, only survivors with relatively long TLs may be involved, and, thereby, the variability in TL can be reduced (Mather et al., 2011). The variability of TL was indeed found to be reduced in lymphocytes, CD45RA-positive T-cells and memory T-cells in healthy oldest-old persons compared to mid-life controls (Halaschek-Wiener et al., 2008).

## TL and Disease

Recently, a hypothesis was proposed that aging trajectory and longevity potential may be “programmed” early in development (Vaiserman et al., 2018). In particular, it is assumed that intrauterine growth restriction (IUGR) can result in a low birth weight and a high risk for metabolic disorders in adulthood, whereas fetal macrosomia and resulting high birth weight can predict an enhanced risk for cancers in adulthood (Vaiserman, 2018). Telomere biology is suggested to play an important role in mediating these programming effects. According to the “fetal programming of telomere biology” hypothesis proposed by Entringer et al. (2018), both the initial TL and the life-course rate of telomere shortening are highly plastic and sensitive to early-life conditions. As a consequence, stress exposures (maternal-placental-fetal endocrine or metabolic disturbances, oxidative and immune/inflammatory stresses, etc.) acting during prenatal and early postnatal development can program the telomere biology in a way that promote cellular senescence and, as a result, lead to accelerated rate of organismal aging. Possibility of developmental programming of initial TL and rate of age-related telomere erosion could be particularly important in the context of aging and aging-associated pathology. According to a two-hit developmental model of accelerated aging (Belsky and Shalev, 2016; Shalev and Belsky, 2016), shorter TL in early life may be associated with poor health status later in life. Building on these ideas, Aviv and Shay (2018) further hypothesized that lengthened telomeres may enhance the risk for disorders linked to increased proliferation rate, including cancer, while shortened telomeres can enhance the risk for pathologies related to a restricted cellular proliferation and tissue degeneration, such as atherosclerosis-associated cardiovascular disorders.

In epidemiological studies, TL was repeatedly found to reflect the risk for aging-related chronic pathological conditions. In patients with coronary heart disease, TLs were found to be significantly shorter than that in controls; moreover, TL was inversely correlated with the severity of this disease (Xu et al., 2019). The convincing evidence was obtained that short telomeres are indicative of higher risk for atherosclerosis and related vascular complications (De Meyer et al., 2018; Herrmann and Herrmann, 2020). Low telomerase activity and short LTLs were shown to be associated with atherosclerotic plaque instability and higher risk for coronary heart disease, myocardial infarction or stroke (Yeh and Wang, 2016; Tian et al., 2019; Zhan and Hägg, 2019). Significant associations were observed between the high rate of age-related LTL attrition and hypertension (Liu et al., 2019b). In a systematic review comprising 119,439 individuals in total, a weak to moderate association between obesity and TL was observed in 39 of 63 included studies; a significant heterogeneity, however, was observed between studies (Mundstock et al., 2015). An association of short LTL with higher risk for developing type 2 diabetes has been also demonstrated; this association, however, was significantly influenced by body mass index (BMI), diabetes type, age, region, and sex (Zhao et al., 2013; Willeit et al., 2014; Wang et al., 2016; Krasnienkov et al., 2018). Short LTL was also shown to be associated with different aspects of metabolic syndrome (Khalangot et al., 2017, 2019, 2020). Accelerated telomere shortening and low telomerase activity were shown to be associated with skeletal pathologies mediated by the age-related abnormal reconstruction of the subchondral bone, such as osteoporosis and osteoarthritis (Fragkiadaki et al., 2020).

Available data on TL in aging-associated neurodegenerative disorders are rather inconsistent. No evidence for shorter TLs in patients with Parkinson's disease was obtained in meta-analysis by Forero et al. (2016a). An evidence for shorter TL in patients with Alzheimer's disease has been provided in another meta-analysis by Forero et al. (2016b). One potential explanation for this could be the enhanced level of oxidative stress in those patients (Levstek et al., 2020). In a more recent study, however, a U-shaped association between the TL and the risk for Alzheimer's disease was demonstrated, with both shorter and longer telomeres associated with an increased risk of this disease in the general population (Fani et al., 2020). Given the uncertainty of findings, the contribution of telomere attrition in the pathogenesis of neurodegenerative disorders is yet to be fully elucidated (Levstek et al., 2020).

Epidemiological associations between TL and risk for cancers are also ambiguous. The tumor cells are known to be able to maintain TL for unlimited growth either by reactivation of telomerase or by a specific recombination-based mechanism (Okamoto and Seimiya, 2019). From this, it is commonly thought that the risk of diseases mediated by increased proliferative activity including most cancers have to be correlated with long telomeres (Celtikci et al., 2020). In fact, however, cancerous cells frequently have paradoxically shorter telomeres relative to those observed in normal tissues (Okamoto and Seimiya, 2019). The evidence that cancers are associated with short telomeres in surrogate tissues (e.g., in blood cells) was provided in a meta-analysis by Wentzensen et al. (2011). The strongest evidence has been obtained for gastric, esophageal, renal and bladder cancers. A significant association between short TL and poor cancer survival has been also demonstrated (Zhang et al., 2015). The association between short telomeres and the risk for cancer was consequently confirmed in other meta-analyzes. In particular, convincing evidence has been obtained for the association of short telomeres with elevated risk of gastrointestinal, head and neck cancers (Zhu et al., 2016). However, in a meta-analysis by Zhang et al. (2017), an association between the TL and the risk of total cancers was found to be marginally positive. Subgroup analyses performed within this meta-analysis showed that such a positive association was stronger for a lung cancer, for men and for studies with more precise methods for DNA extraction and TL measurement. In conclusion, the inconsistency in the effects of TL on cancer outcomes can be likely explained by the variable measurement methods. Therefore, the standardization of measurement and reporting of TL is required in cancer epidemiology before the prognostic value of TL might be accurately evaluated (Adam et al., 2017).

From the research findings above, it can be concluded that epidemiological associations between TL and age-dependent pathological conditions are often inconsistent and a mechanistic understanding of these associations is still lacking. Remains uncertain whether age-related telomere shortening is a cause or merely a consequence of aging-associated diseases (De Meyer et al., 2018). Moreover, despite convincing evidence has been provided for links between TL and specific age-related diseases, no significant relationship was found between the TL and syndrome of aging-related physiological decline (frailty), a geriatric clinical syndrome characterized by marked vulnerability to adverse health outcomes (Zhou et al., 2018; Araújo Carvalho et al., 2019).

## TL and Mortality

Numerous epidemiological findings indicate that short telomeres are associated with higher morbidity and all-cause mortality (Wang et al., 2018) and TL appears to be a better predictor for survival than chronological age (Adwan Shekhidem et al., 2019). For example, in the Swedish Twin Registry study, individuals with the shortest LTL quartile had 44% higher risk of all-cause mortality than those with the longest quartile (Wang et al., 2018). Moreover, the meta-analysis of all eligible studies performed by the authors (121,749 persons with 21,763 deaths in total) indicated that individuals with the shortest LTL quartile had 26% higher risk of all-cause mortality compared to those with the longest quartile, although substantial between-study heterogeneity has been revealed. In an individual-participant-data meta-analysis of 2 large prospective cohort studies from Europe and the U.S. conducted by Mons et al. (2017), age-adjusted hazard ratios for the shortest LTL quintile compared to the longest were found to be higher by 23, 29, and 10% for all-cause mortality, cardiovascular mortality, and cancer mortality, respectively. Likewise, in the Ludwigshafen Risk and Cardiovascular Health (LURIC) study, patients in 2-4 LTL quartiles had 18% lower hazard ratio for all-cause mortality and 17% lower hazard ratio for CVD-mortality when compared to those in the 1st quartile (Pusceddu et al., 2018). The associations between TL and mortality were, however, not consistent across studies. For example, in a systematic review by Mather et al. (2011), a significant correlation between shortened TL and increased risk of mortality was observed in 5 of 10 studies included in the analysis, while it was absent in 5 others. All these studies, however, were performed with a cross-sectional design, which do not allow an inference of causality. Given this, and also because of a very high inter-individual variability of TL in same-age individuals, longitudinal research of intra-individual rates of age-related decline in TL is likely a more useful design than the cross-sectional one in studying the relationship between TL and mortality. However, this link was investigated with a longitudinal design in few studies only. In the study by Epel et al. (2008), short baseline LTL was associated with 2.3-fold higher risk for mortality from cardiovascular disease in females; in male, age-related LTL shortening, but not baseline LTL, was associated with 3-fold higher risk for cardiovascular mortality. In studying 3259 adults of European ancestry residing in the U.S., short telomeres were associated with an increased risk of non-cancer mortality as individuals approached the upper boundary of their longevity (Arbeev et al., 2020). The longitudinal changes in LTL in relation to all-cause, cardiovascular, and cancer mortality were observed in 247 elderly Swedish males (Yuan et al., 2018). Short LTLs at baseline were strongly associated with mortality risks, with a 40–70% enhanced risk of all-cause mortality, and a 2-fold enhanced risk of cancer mortality. The association between TL and mortality, however, has not been confirmed in several other studies. For example, in the large population-based follow-up research by Martin-Ruiz et al. (2005), TL in blood monocytes had no predictive power for age-related morbidity and mortality in the oldest-old aged 85 years and over. In addition, whereas no association was observed between LTL and overall survival or death from any specific cause, a significant association between LTL and self-reported health status was found in both cross-sectional and longitudinal data (Njajou et al., 2009), indicating that LTL may not be a strong predictive biomarker for prognosis of survival in older individuals, but it may be a potentially useful biomarker of healthy aging.

## TL and Other Biomarkers of Aging: Whether They Measure The Same Processes?

When discussing the applicability of LTL as a reliable biomarker of aging, the comparison of LTL and other biomarkers of aging from the same individuals may certainly provide valuable information. In this context, the comparison of LTL with biological age measure such as DNA methylation-based epigenetic clock [DNA methylation (DNAm) age], which is currently regarded as the most precise estimation of biological age (Bell et al., 2019; Levine, 2020; Salameh et al., 2020), seems to be particularly valuable. It is yet questionable whether LTL and DNA methylation change in tandem or contribute independently to the prediction of biological age. To answer this question, several studies have been recently conducted. Evidence that TL and epigenetic clock estimates are independent predictors of chronological age and mortality risk was obtained in the study by Marioni et al. (2018) performed in two Scottish cohorts aged from 70 to 90 years. In both cohorts studied, combined whole-blood TL and DNAm age explained more variance in age than each of them individually. In a combined cohort analysis, TL and DNAm age explained 2.8 and 28.5% of the variance in age, respectively, and jointly they explained 29.5%. Also in a combined cohort, one standard deviation increase in a baseline DNAm age was associated with a 25% increased mortality risk (*p* < 0.001) while in the same model, one standard deviation increase in a baseline TL was independently associated with an 11% reduced mortality risk only (*p* = 0.05). In addition, weak, non-significant correlations have been observed between the estimates of epigenetic clock and TL. In older adults, the whole-blood DNAm-based mortality risk scores were shown to be strongly associated with TL; they, however, demonstrated much stronger associations with all-cause mortality than the TL-based ones (Gao et al., 2018). More recently, a significant correlation between DNAm age and chronological age was found in the study by Banszerus et al. (2019). However, no evidence of either association between the relative LTL and DNAm age or between LTL and the DNAm age acceleration was observed in the studied cohort, suggesting that LTL and DNAm age measure different aspects of biological age. In the subsequent study, a weak but significant inverse relationship between DNAm age acceleration and relative LTL was shown (Vetter et al., 2019). The authors concluded that DNAm age is a biomarker of aging phenotypes, which are not (only) linked to pathways associated with mitotic age as measured by relative LTL. In this context, it should be noted, however, that TL is not the only unique indicator of mitotic aging. Indeed, as it was shown by Lowe and co-authors, epigenetic clocks also can be indicative of mitotic age, as demonstrated by advanced ticking in cultured cells that were forced into replicative senescence but no change in those cells forced into senescence via DNA damage (Lowe et al., 2016). Recently, a novel DNAm-based TL estimator was introduced by Lu et al. (2019). This epigenetic biomarker was developed by regressing measured LTLs on the blood methylation data (140 CpGs) for 2,256 individuals. This estimator correlated negatively with age in different tissues and in various cell types and outperformed LTL measurements generated using classical Southern Blot-based approach when predicting morbidity and mortality.

In the Belsky et al. (2018) study, eleven different methods were used to quantify biological aging in the same individuals. These methods included TL and the rate of TL shortening, and also three epigenetic-clocks and their ticking rates, and three biomarker-composites. The associations between these measures and aging-related outcomes such as cognitive decline, physical functioning, and also subjective signs of aging, including aged facial appearance were determined. The 71–cytosine-phosphate-guanine epigenetic clock and composite biomarkers were found to be associated with aging-related outcomes studied, however, the effect sizes were modest only, and TL-based measure was not associated with any of outcomes. Moreover, unexpectedly, poor agreement was observed among all applied measures of biological aging. The authors assumed that different methods of quantifying biological aging could not measure the same aspects of the aging process.

**Figure 5:**
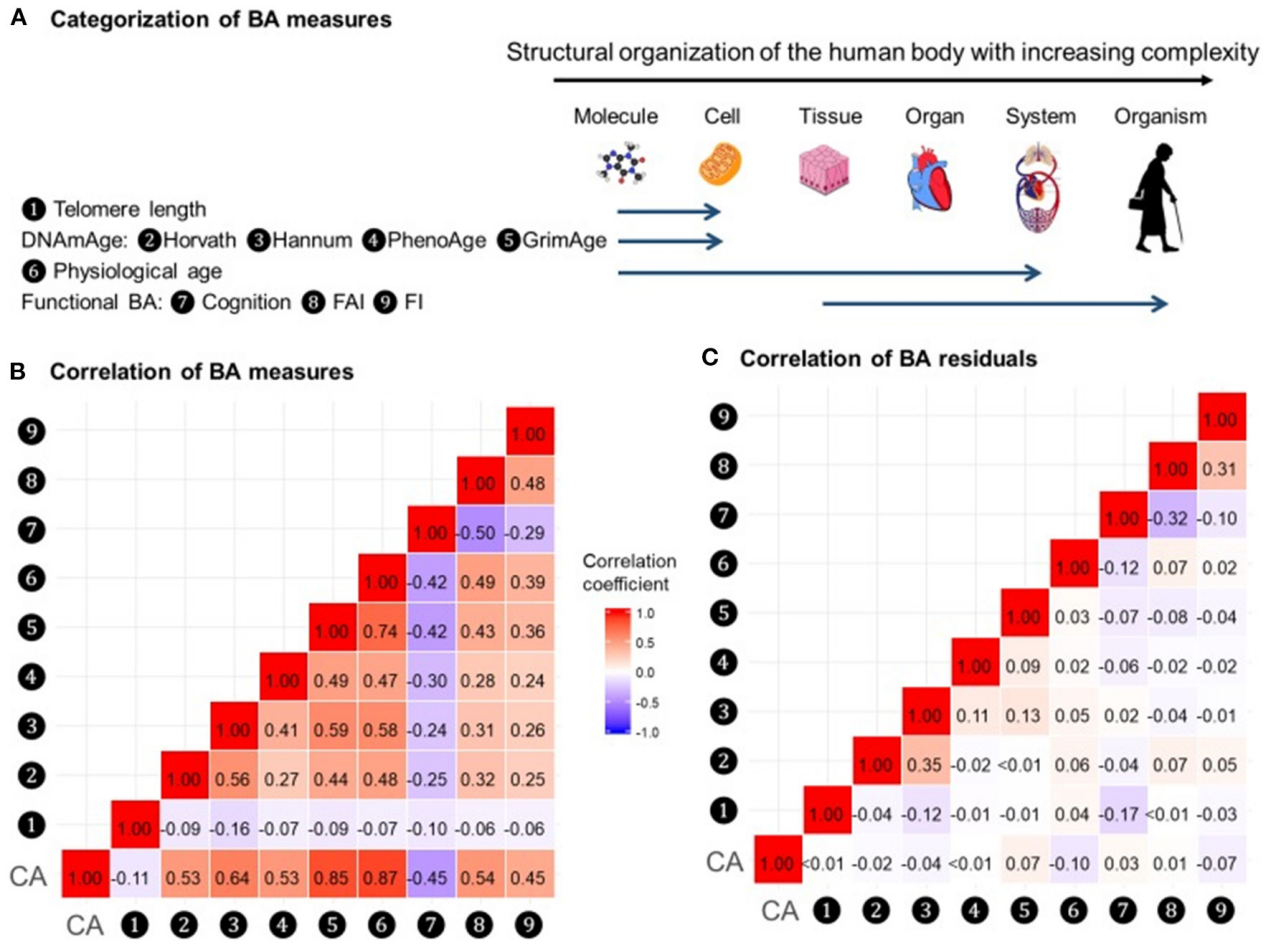
Correlations of BAs in 288 individuals (612 complete measurements). A total of 612 complete measurements assessed from 288 individuals were included to estimate the correlations of BAs. BAs were broadly categorized into four groups according to the main biological structural levels where the BA measurements were implemented **(A)**. We estimated the repeated-measure correlation coefficients between BAs and between BA residuals and illustrated the correlation coefficients in heat maps **(B,C)**. Red and blue tiles represented positive and negative correlations, respectively; color density indicated the magnitude of correlation coefficients. All BAs were correlated to varying degrees **(B)**. After regressing out CA from BAs, most of the original correlations were attenuated **(C)**. BA, biological age; DNAmAge, DNA methylation age estimator; FAI, functional aging index; FI, frailty index; CA, chronological age. The figure and its legend are reproduced from the article by Li et al. (2020) distributed under the terms of the Creative Commons Attribution License. Copyright © 2020, Li et al.

**Figure 6:**
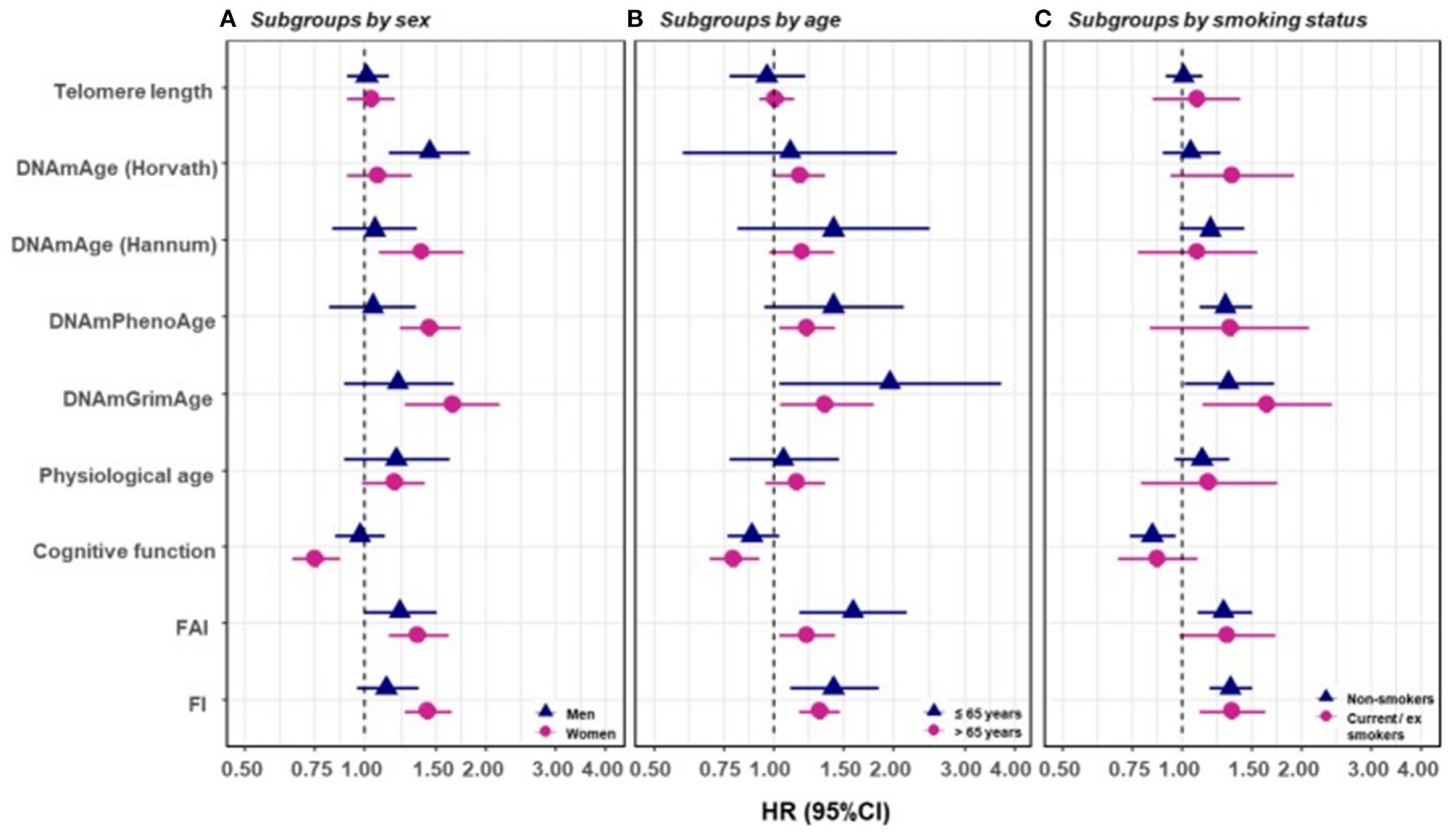
Survival analyses of baseline BAs with the risk of all-cause mortality in subgroups classified by sex, baseline smoking status, and baseline age (one-BA models). A total of 845 individuals were included to estimate the mortality associations of BAs in subgroups. We used Cox regression models to estimate the change in mortality risk associated with a one-SD increment of the respective BA at baseline assessment (one-BA models). All models controlled for sex, educational attainment, smoking status, and BMI, stratified by participants' birth year, and accounted for left truncation and right censoring. Attained age was used as the time-scale and thus age was inherently adjusted for. BA-mortality associations were illustrated in the forest plot **(A–C)**, in which points and horizontal lines denoted HRs (95% CIs) and point shapes and colors represented subgroups. The associations of BAs with mortality risk were generally stronger in women (except for Horvath DNAmAge and physiological age), more pronounced in the younger individuals (except for Horvath DNAmAge, physiological age and cognitive function), and a bit stronger in current or ex-smokers (for Horvath DNAmAge and DNAmGrimAge). BA, biological age; DNAmAge, DNA methylation age estimator; FAI, functional aging index; FI, frailty index; CA, chronological age; HRs (95%CIs), hazard Ratio (95% Confidence Interval). The figure and its legend are reproduced from the article by Li et al. (2020) distributed under the terms of the Creative Commons Attribution License. Copyright © 2020, Li et al.

In a recent study by Li et al. (2020), nine measurements of biological age (Figure 5A) were assessed in a 20-year follow-up in 845 individuals from a Swedish population-based cohort. All measurements of biological age were more or less correlated with one another. Interestingly, the correlations among LTL and other biological age measurements were lower than among all other measurements (Figure 5B). The correlation became greater if the biological age residuals constructed by regressing out the chronological age-related part from respective biological age (commonly referred to as indices of age acceleration in the DNAm age-related studies) were used (Figure 5C). The largest effects were shown for estimators of methylation age (GrimAge) and the frailty index. In predicting the mortality of the study participants, two estimators of methylation age (Horvath and GrimAge) and the frailty index remained predictive in joint models, indicative of the complementarity between them, and all biological age measurements except for LTL have been related to mortality risk independently of chronological age (Figure 6).

Basing on the findings above, it can be concluded that measurements of biological aging conducted either with TL-based approaches or with other approaches, such as the DNA methylation-based epigenetic clock, could measure different aspects of the aging process. Therefore, it seems reasonable to use them together rather than separately.

## Using TL in Human Studies: Analytical and Methodological Challenges

### Analytical Aspects

**Figure 7:**
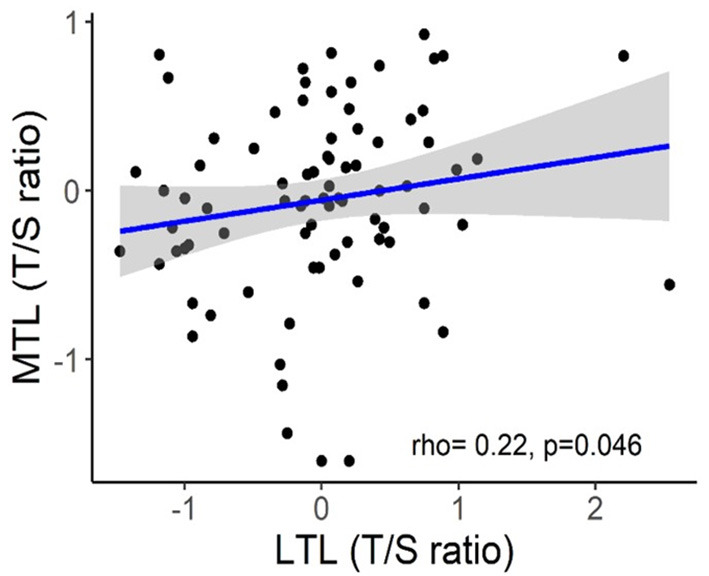
Correlation between skeletal muscle TL (MTL) and LTL. The figure and its legend are reproduced from the article by Hiam et al. (2020) distributed under the terms of the Creative Commons Attribution License (CC BY 3.0). Copyright © 2020 Hiam et al.

The implementation of LTL as routine marker into clinical practice is currently hampered by several unresolved pre-analytical and analytical issues (Semeraro et al., 2020). One serious problem is considerable inter-individual variability of LTLs which was repeatedly observed in molecular epidemiological studies across multiple populations and which may substantially complicate the interpretation of individual data (Bodelon et al., 2014). For example, in the Factor-Litvak et al. (2016) study, LTL range was 7.01–11.6 kb in the newborns, 6.19–9.81 kb in their mothers (age range, 17–42 years), and 5.83–9.88 kb in fathers (age range, 17–56 years). Another important issue is that TL may substantially vary across various tissue types. There is also evidence that TL may vary even within the same organ depending on the site of sampling (Semeraro et al., 2020). However, in some cases, TLs may be correlated in different organs even despite these differences. Correlation between TLs in leukocytes and skeletal muscles was demonstrated in recent study by Hiam et al. (2020) conducted in healthy individuals across a wide age range (18–87 years). Those subjects who had long (or short) TL in one tissue also tended to have long (or short) TL in another (Figure 7). This correlation between TLs in leukocytes and other somatic tissues within an individual may be likely explained by high inter-individual TL variation, which is already evident at birth (Factor-Litvak et al., 2016). This variation was found to be about three times larger than that in TL within the individual's somatic tissues (Aviv and Levy, 2019). There are, however, differences in TLs amongst somatic tissues primarily related to the replicative history of stem/progenitor cells in these tissues.

**Figure 8:**
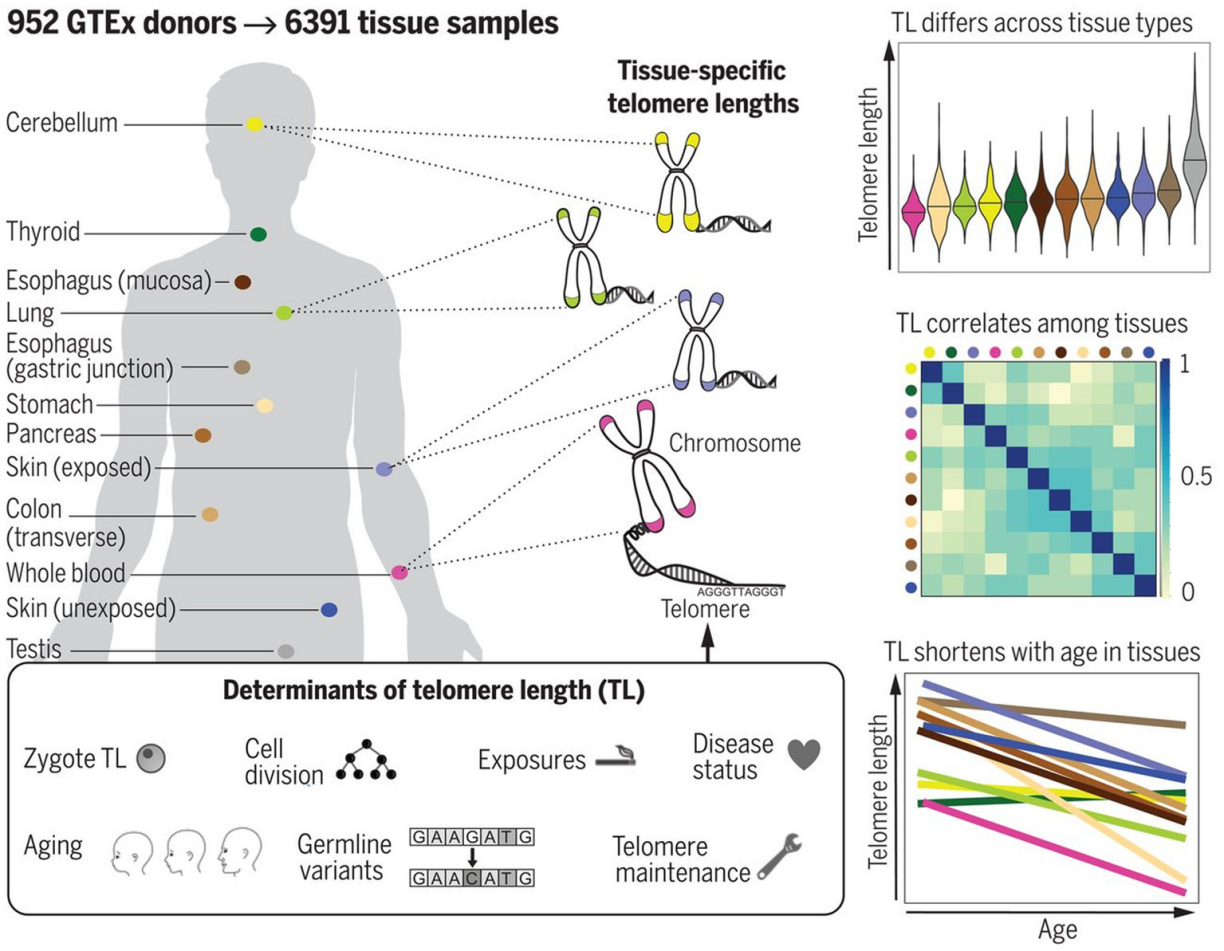
TL in human tissues. Using a Luminex-based assay, TL was measured in DNA samples from >25 different human tissue types from 952 deceased donors in the GTEx project. TL within tissue types is determined by numerous factors, including zygotic TL, age, and exposures. TL differs across tissues and correlates among tissue types. TL in most tissues declines with age. The figure and its legend are reproduced from the article by Demanelis et al. (2020) with permission from AAAS (License Number 4910920603987). Copyright © 2020 The Authors.

The most reliable information in this respect would be certainly provided from studies performed with cadaver specimens. Variability of TL in different cadaver tissues from the same dead human donors has been, however, investigated in few studies to date. In the study by Dlouha et al. (2014), TL was studied in tissues obtained from deceased donors aged from 29 weeks to 88 years. The relative TL (rTL, telomere repeat abundance in a DNA sample relative to a standard sample) was significantly higher in blood compared to liver, brain, heart, muscles and skin and moderately higher compared to spleen and mucosa, while no differences were obtained with adipose and renal tissues. The longest rTL was found in leukocytes (1.34 times longer than the reference TL) and kidney (1.15), and the shortest one was observed in the skin (0.48), liver (0.57), and brain (0.58). These inter-tissue differences were likely due to differences in the proliferation rate and different life spans of the various cell types, and also in oxidative stress levels in these tissues. These results, however, could be confounded by the small sample size and also by the variable health status of donors. Convincing evidence supporting differences in TL amongst human tissues has been provided in a more recent research by Demanelis et al. (2020). In this large-scale study, rTL has been measured during the Genotype-Tissue Expression (GTEx) project in more than 25 tissue types from 952 deceased donors (age from 20 to 70). Generally, rTLs were shown to be positively correlated between tissues. The whole-blood rTL was found to be a proxy for rTLs in most of the tissues studied. rTL varied across tissues and was shown to be shortest in the whole blood and longest in testicular tissue. In most of the tissues studied, except for the testis and cerebellum, rTLs were inversely associated with age; this association was found to be strongest in tissues with shorter average rTL. Moreover, importantly, evidence was provided that TL-associated genetic variants can affect expression of nearby genes and that TL may mediate effects of age on gene expression in various tissues. In addition, components of telomerase have been found to be more highly expressed in testis than in any other tissue. For schematic representation of the main findings from the study by Demanelis et al. (2020), see Figure 8.

Another analytical issue in human studies is that TL is most commonly determined in peripheral blood leukocyte samples. This cell source was chosen for TL analysis because it allowed avoid invasive procedures. However, it still remains questionable whether findings in circulating leukocytes can be generalized to other tissues. There is evidence from several studies that TL in leukocytes reflects systemic TL in other tissues. For example, TLs were found to be strongly correlated in adult tissues such as leukocytes, skin, skeletal muscle and subcutaneous fat, and rates of telomere shortening were similar in these tissues (Daniali et al., 2013). It indicates that TL in circulating leukocytes may be a good surrogate marker for TL in other somatic cell types. There is, however, some evidence that this could be not true for certain tissues (Thomas et al., 2008). It remains far from clear whether correlations exist between TLs in leukocytes and in tissues with low proliferative activity, such as neurons (Mather et al., 2011).

**Figure 9:**
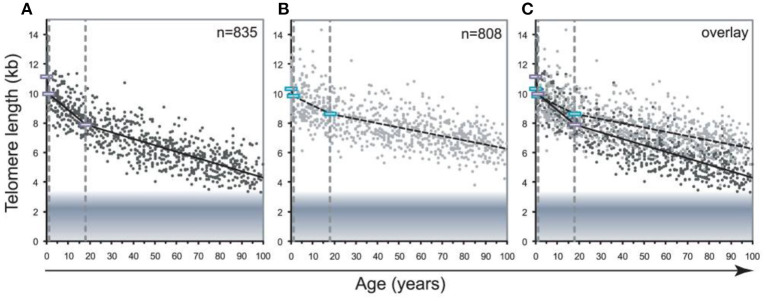
Decline in telomere length with age differs between lymphocytes and granulocytes. The median telomere length in nucleated blood cells from 835 healthy individuals ranging from birth (umbilical cord blood) to 102 years of age were measured by flow FISH. The results were used to calculate the telomere attrition over time using linear regression in three age segments. **(A)** Median telomere length in lymphocytes (black dots). **(B)** Median telomere length in granulocytes (gray dots). Breakpoints in the piece-wise linear regression lines are marked by rectangles and the three age groups are marked by dotted vertical gray lines at 1 and 18 years. On average 8 individuals were tested per age-year. **(C)** At any given age, a wide range of telomere length was observed and the decline in telomere length with age in lymphocytes was more pronounced than in granulocytes. The shaded area represents the estimated length of subtelomeric DNA. Note that in older individuals, on average only 1–2 kb of telomere repeats were present in lymphocytes. The figure and its legend are reproduced from the article by Aubert et al. (2012a) distributed under the terms of the Creative Commons Attribution License. Copyright: © 2012 Aubert et al.

One more important methodological problem is that blood leukocytes are highly heterogeneous cell population which includes various cell types, such as lymphocytes, monocytes, granulocytes, etc. The composition of this cellular population is known to be highly variable, even in healthy persons, depending on various stress exposures (Semeraro et al., 2020). Different stressors may trigger a redistribution of leukocytes from immune reservoirs to the circulation and certain peripheral tissues (Dhabhar et al., 2012). This is an important issue given the fact that TLs differ in leukocyte subtypes isolated from the same donors. For example, in the Lin et al. (2010) study, the highest level of the telomerase activity and longest TLs were observed in B cells while CD4+ T cells had slightly higher telomerase activity and similar TLs than CD8+CD28+ T cells. Moreover, transient expression of telomerase can occur as a result of the antigen-induced lymphocyte stimulation. Although the telomerase activity is low enough in resting human T lymphocytes, it was shown to be transiently upregulated upon antigen presentation (Huang et al., 2017). In addition, pronounced differences exist in the rate of age-related telomere attrition in different tissues and cell types. For example, the decline in TL with age was found to be far more pronounced in lymphocytes than in granulocytes (Aubert et al., 2012a) (see Figure 9 for illustration).

Another important point is that, for each particular individual, LTL is a very dynamic parameter reflecting transient changes in the immune system which have nothing to do with aging. Indeed, inflammatory conditions may induce proliferation of leukocytes in bone marrow. Therefore, the proportion of newly released leukocytes may be enhanced in such conditions in total leukocyte population. Newly differentiated (naïve) leukocytes have TLs similar to those of hematopoietic stem cell progenitors, whereas much shorter telomeres are in mature leukocytes (Kimura et al., 2010). These processes can likely influence LTL. Since LTLs are clearly affected by the leukocyte turnover, it makes it difficult to determine whether variations in LTL can be attributable to a blood-sample leukocyte composition or to a particular condition of interest (Hastings et al., 2017; Krasnienkov et al., 2018). It is also necessary to take into account that age-related variation in LTL could reflect different histories of the individual's immune responses under differing environmental conditions. As the proliferative potential is compromised and TLs are shorter in memory compared to naïve cells (Weng et al., 1995), LTL might be relatively shorter when a large proportion of memory CD8+ T cells is present in the leukocyte sample. Evidence is obtained that, for a given age, those individuals whose blood contains comparatively less naïve CD8+ T cells and more memory CD8+ T cells may display relatively shorter LTLs and more old DNAm age (Chen et al., 2017). Thus, since age-related TL attrition in adult T cells reflects the history of their antigen-mediated induction, this process may mirror, at least partly, the aging of the immune system of an individual.

The clonal hematopoiesis of indeterminate potential (CHIP) also may be a factor potentially influencing age-related dynamics of TL. CHIP is a common aging-related phenomenon in which hematopoietic stem cells (HSCs) and other early progenitors of blood cells contribute to the formation of genetically distinct subpopulations of blood cells in aging humans (Jaiswal and Ebert, 2019). Recently, it has been found in epidemiological studies that human aging is commonly associated with an increased frequency of somatic mutations in the hematopoietic system. In this process, particular mutant cells receive a competitive advantage allowing their clonal expansion, a process referred to as clonal hematopoiesis (Evans et al., 2020). Thus, each of such arising subpopulations is clonally derived from a single founder progenitor cell, thereby representing a genetic clone of this founder. These cell subpopulations are characterized by shared unique DNA mutations which occur most commonly in the transcriptional regulators DNMT3A, TET2, and ASXL1 (Jaiswal and Libby, 2020). CHIP was repeatedly found to be associated with a variety of adverse health outcomes, including hematological cancers, and also with a significantly elevated risk of atherosclerotic cardiovascular disorders which are a leading cause of death in the elderly (Evans et al., 2020; Jaiswal and Libby, 2020). The incidence of clonal hematopoiesis was observed to rise dramatically with individual's age. Clonal hematopoiesis was found in only 1% of persons younger than 50 years of age but it was evident in about 10% of persons older than 65 years of age), and about 20% of octogenarians were shown to be CHIP carriers (Genovese et al., 2014; Aviv and Levy, 2019).

The dynamics of TL in the cellular components of “hemothelium” (a vascular endothelium as a single entity) was proposed to be a central factor in the genesis of both CHIP and atherosclerosis (Aviv and Levy, 2019). This hypothesis is primarily based on findings from the whole genome sequencing and genome-wide-association analyses indicating that those subjects who display the features of CHIP have relatively short LTLs and often harbor a certain variant of the gene encoding telomerase (Zink et al., 2017). Moreover, CHIP was found to be common in patients with dyskeratosis congenita, a disease resulting from mutation in TL maintenance genes, including those that encode telomerase subunits and thereby causing critically short telomeres. In addition, the prevalence of CHIP was shown to be higher in the general population in subjects with relatively short LTLs, including males, who are known to have shorter LTLs than females, and elderly, who have shorter LTLs than younger individuals. Finally, age-associated rise in the prevalence of CHIP also provides supporting evidence. According to this hypothesis, age-related telomere shortening influences all proliferative tissues, but particularly the hematopoietic system characterized by a high proliferation rate. With age, TLs in hematopoietic stem/progenitor cells become progressively shorter, gradually approaching a “telomere brink,” i.e., critically short TLs thereby compromising the function of these cells and increasing the risk of death in the near future (Steenstrup et al., 2017; Aviv and Levy, 2019). If these considerations are correct, then it can be assumed that emergence of hematopoietic clones with increased replicative potential due to *de novo* mutations in hematopoietic stem/progenitor cells may enable several individuals to postpone reaching the critically shortened telomere threshold (“telomere brink”).

### Methodological Aspects

There are also some methodological issues that hamper a wider application of TL analysis in clinical and epidemiological research. An important limitation of most existing TL measurement approaches is their reliance on producing a measure of average TL, which is not representative of the mechanisms linking TL to aging; indeed, the senescence process can be triggered by a single shortest telomere (Hemann et al., 2001). Some techniques have been developed to measure TL; each of these approaches, however, has distinct advantages and disadvantages. A comprehensive overview of the most useful TL measurement methods can be found in the review by Lai et al. (2018).

The Terminal Restriction Fragment (TRF) method is regarded as the gold standard for TL measurement. This method uses the Southern blotting or in-gel hybridization with a labeled probe specific for telomere DNA, providing an average TL value for the total cell population (Jenkins et al., 2017). The applicability of this technique is, however, substantially limited by the requirement of large (about 3 μg) amounts of DNA for analysis. Another important limitation of the TRF technique is that restriction enzymes used in this method lead to the inclusion of sub-telomeric DNA that is contiguous to the telomere, thereby resulting in an overestimation of the true TL (Montpetit et al., 2014). Moreover, this assay presents a relatively laborious procedure. In addition, very short telomeres (up to 2 kb) are difficult to detect with this method. Although reproducibility of TRF method within the same laboratory is rather good, the data obtained cannot be easily compared between different laboratories. The possibility of inter-laboratory comparability, however, emerged after the appearance of commercial kits for the TRF assay on current market.

Average cell TL and other TL-related parameters may be determined by the fluorescent *in situ* hybridization (FISH) technique by using flow cytometry (flow FISH) or digital microscopy (quantitative FISH, Q-FISH) (de Pedro et al., 2020). This technique is highly accurate and allows detect even subtle changes in TLs. It, however, requires fresh cell samples and is technically demanding. Recently, high-throughput Q-FISH method was developed for examining many variables of individual telomeres. This method represents the combination of high-throughput imaging and software workflows. It allows simultaneous collection of a large number of telomere-associated variables in a large number of peripheral blood mononuclear cells. It should be noted, however, that although FISH-based methods produce highly reliable results, they are quite labor intensive and require expensive equipment. Importantly, the frequency of short telomeres (<3 kbp) can be accurately evaluated with this method. This is an important point because relative frequency of shortest telomeres which may trigger a cell cycle arrest is a parameter critical for cell viability and chromosome stability, and it is highly associated with mortality (Hemann et al., 2001). Due to these properties, this technique may be useful in epidemiological and clinical studies. Furthermore, reliable measurement of shortest telomeres with this method could provide new opportunities in assessing biological age.

Recently, a new method called Telomere Shortest Length Assay (TeSLA) was developed for measuring the distribution of the shortest telomeres in heterogeneous telomere backgrounds (Lai et al., 2017). This method improves the efficacy of TL measurements after Southern blot analysis by using specific image-processing software aimed to automatically detect and annotate band sizes, and also calculate average TLs and percentages of shortest telomeres. With TeSLA, it is possible to detect telomere dynamics in a range from <1 to ~18 kb in both normal aging processes and in telomere-related disorders in humans. In particular, TeSLA is capable of measuring different cellular subpopulations in peripheral blood mononuclear cells, e.g., CD28(–) T cells, which have a limited capability for cell division, shorter telomeres, and more high rate of telomere shortening relative to other subtypes of mononuclear cells (Weng et al., 2009). Therefore, this method can provide an opportunity to identify critically short telomeres in specific subsets of immune cells which are known to be largely contributed to age-related decline of immune function.

The quantitative polymerase chain reaction (PCR) assay is most widely used now. This assay is easy to perform, allows high throughput and requires small amount of DNA (Lin et al., 2019). Due to its high throughput, this technique has been often applied in large population studies. This method allows determine the number of copies of telomeric repeats (T) compared to a single copy gene (S) and its results are expressed as a T/S ratio. However, information about the distributing long and short telomeres and also regarding the differences between individual cells and chromosomes cannot be obtained with this approach. Moreover, the variability of data obtained with this technique may be substantial and exceed 10% within and between samples (Aubert et al., 2012b).

Concluding, although many innovative technological approaches were developed to measure TL, there still are substantial inter-laboratory variations and the results obtained by one method can substantially differ from those obtained by other which makes it difficult to use them in epidemiological and clinical studies (Dagnall et al., 2017). It should also be noted that accuracy and reproducibility of TL measurements depend, along with assay procedures, on many other factors including the sample collection, processing and storage, DNA extraction, etc. (Lin et al., 2019). Therefore, further efforts should be made to optimizing these techniques in order to improve their sensitivity, repeatability and throughput.

### TL: A Single Biomarker or a Part of Composite Biomarker Panel?

Since the discovery of the phenomenon of age-related telomere attrition, TL has attracted a great deal of attention in gerontological research as one of the most promising biomarkers of aging. However, despite well-founded theoretical background for that, the available empirical evidence is rather contradictory. Therefore, the measurement of LTL is not yet widely-used in routine clinical diagnostics. In most epidemiological and clinical studies, only weak relationships were observed between TL and age-sensitive indices of physical functioning such as blood pressure, grip strength and lung function; more strong association was found for indices of cognitive performance, although the results were not unequivocal as well [for review, see Mather et al. (2011)]. Results from these studies indicate that TL does not reflect underlying aging processes and therefore cannot be considered as universal marker of biological aging. Many authors, however, acknowledge that biomarkers of aging can change over the life course. Moreover, a single biomarker could not sufficiently reflect the aging process across various biological systems. Contradictory findings from these studies could be likely explained, at least partly, by small sample sizes (and, consequently, by limited statistical power to detect associations) and also by narrow age ranges investigated (Der et al., 2012). Moreover, a cross-sectional design is an important limitation of most these studies. Such design may provide only limited inferences regarding causality.

In many studies, TL was examined as a single measure of aging rate. It is an important point because a question still remains whether TL can be used as a single biomarker of aging or as a part of composite biomarker panel only. To answer this question, Der et al. (2012) conducted a large community-based prospective cohort study in a Scottish population. In this research, two composites from the measures of functioning have been formed by principal components analysis, one of which included the TL and the other did not include it. Both these composite biomarkers of aging have been found to be better predictors of overall health outcomes than chronological age. There were, however, several differences between these two composites. TL was shown to be significantly associated with age and with eight measures of physical and cognitive functioning known to be related to normal aging. These measures included indices of physical functioning (pulse pressure, lung function and grip strength), cognitive functioning (a general mental ability test and a four-choice reaction time scores) as well as overall measures of health status (registered disability, self-rated health and the total number of chronic conditions). In most cases, TL added predictive power to that of age though, according to the authors, it was found to be not nearly as good a predictor overall. More specifically, when adjusted for age, the association with TL remained statistically significant in five out of eight cases, thereby indicating that it may add predictive power over and above that of age. It, however, accounted for a very small proportion of the effect of age only and therefore does not satisfy itself the strict criterion of being a better predictor than age *per se*. These findings, collectively, suggest that TL, as a single biomarker, does not quite satisfy the criteria commonly applied for biomarkers of aging, but it does add predictive power to that of chronological age. The authors emphasize, however, that the fact that TL only modestly contributed to the composite biomarker of aging can lead to misleading interpretation of the study results, depending on whether they are used for the prediction or explanation of biological aging. Indeed, TL operates at a lower (cellular) level of the biological hierarchy compared to other components operating at various systemic levels. If telomere attrition was a part of the causal process ultimately resulting in an age-associated functional decline at higher levels, then adjusting for these higher level measures of functioning may likely lead to underestimating the effect of TL. In a more recent study by Hastings et al. (2019), composite measures of biological aging along with TL were quantified. All these measures correlated with participants' chronological ages. Three biological aging composite biomarkers were correlated with one another, but none of them was correlated with TL. The authors concluded that TL measures different aspects of the aging process as compared to the patient-level physiological biomarker composites. Moreover, effect sizes for these measures tended to be larger as compared to TL. However, importantly, marginal increases in the effect sizes were observed when TL was integrated into these biomarker composites compared to indices constructed without TL.

Summarizing the results of these studies, it can be stated that the validity of LTL as a single measure of aging rate and as a prognostic tool in clinical settings remain questionable. Indeed, since aging is an extremely complex multivariate process involving multiple molecular pathways operating at many levels of the functional organization, it unlikely may be evaluated with a single biomarker such as TL. Thus, the composite measures definitely have larger predictive value in assessing the aging rate than single measures, including the TL-based ones.

## Discussion

During past decades, TL is recognized as one of the most suitable biomarkers of aging. This is because telomeres are well-known to be critically implicated in cellular aging; moreover, many observations indicate that telomeres tend to shorten with age and that accelerated telomere shortening is a sign for many aging-associated pathological conditions. Therefore, LTL is commonly used as conventional biomarker of aging now. However, findings from available epidemiological studies on the links between TL and age-related diseases and mortality are rather inconsistent and contradictory. Moreover, since these associations were observed mostly in cross-sectional studies, no causal inferences can be made. Furthermore, individual LTL is apparently a very dynamic parameter reflecting changes which are often transient (e.g., following induction of immune responses) and have nothing to do with aging process *per se*. It is still far from clear, whether change in TL is a cause or effect of aging (Turner et al., 2019). Longitudinal designs should be certainly used in future research to prove causality (Chen et al., 2011; Hastings et al., 2017).

Based on these considerations, increased doubts are currently expressed by several authors concerning whether TL really plays a causal role not only in cellular senescence but also in aging of multicellular organism and, accordingly, whether LTL could serve as reliable biomarker of aging (Mather et al., 2011; Der et al., 2012). Many research findings do not confirm that TL meets the main criterion established by the American Federation for Aging Research for a biomarker of aging (Johnson, 2006). Indeed, in many studies, TL was not a better predictor of age-dependent functional declines, morbidity and mortality than chronological age. Moreover, results obtained in investigating these relationships may be substantially biased due to mortality selection in older populations. Furthermore, LTL is largely dependent on the blood-sample leukocyte composition. In addition, it is still not clear whether LTL is a reliable surrogate marker for TL changes in other body tissues, particularly in those with low proliferative activity (e.g., central nervous system), which are currently recognized as main drivers of the aging process. Nevertheless, despite these doubts, and even although epigenetic age-based approaches becomes increasingly favored in the aging research, TL remains the most widely used molecular biomarker of aging now. Innovative approaches, such as single-cell TL measuremens (Wang et al., 2013), techniques aimed at the identification of critically short telomeres (Serakinci et al., 2019) and DNA methylation-based methods of TL estimation (Lu et al., 2019) are being developed to improve sensitivity, repeatability and throughput of methods used for determining TL.

An important point in the context discussed is that, since aging is an extremely complex phenomenon involving multiple pathways and operating at various levels of the biological organization of a living system, it can hardly be accurately measured with a single biomarker. Different measurements of biological age apparently measure different aspects of the aging process. Therefore, estimates of biological age obtained with different measuring approaches may not coincide with each other. Considering this, it is reasonable to assume that TL (if included) may improve the predictive power of composite measures of biological age, while its use as a single biomarker of aging may be questionable in many cases. Indeed, each individual measure included in the composite score would likely point to different aspects of aging process, such as the developmental program (DNA methylation), replicative history of a cellular lineage (TL), environmental stress (mitochondria), etc. (Notterman and Schneper, 2020). Various measures can complement each other thereby improving the predictive power of the composite measure. These limitations need to be considered and challenges need to be addressed before wider implementation of TL as an established biomarker of aging in epidemiological research and clinical trials.

## Author Contributions

AV and DK contributed equally to the drafting, editing, and final composition of the manuscript. All authors contributed to the article and approved the submitted version.

## Conflict of Interest

The authors declare that the research was conducted in the absence of any commercial or financial relationships that could be construed as a potential conflict of interest.
